# Peer Victimization Experienced by Children and Adolescents Who Are Deaf or Hard of Hearing

**DOI:** 10.1371/journal.pone.0052174

**Published:** 2012-12-19

**Authors:** Maartje Kouwenberg, Carolien Rieffe, Stephanie C. P. M. Theunissen, Mark de Rooij

**Affiliations:** 1 Institute of Psychology, Leiden University, Leiden, The Netherlands; 2 Dutch Foundation for the Deaf and Hard of Hearing Child, Amsterdam, The Netherlands; 3 Department of Otorhinolaryngology and Head & Neck Surgery, Leiden University Medical Center, Leiden, The Netherlands; The University of Queensland, Australia

## Abstract

Victimization is a relatively common, yet serious problem, with potentially severe consequences for children's psychosocial and academic functioning. Children who are Deaf or Hard of Hearing (DHH) may be at a higher risk for victimization than hearing children. The aims of the present study were to compare DHH and hearing children on i) self-reported experiences of victimization and ii) associations between victimization, parental- and child variables. In total 188 children (mean age 11;11 years) from the Netherlands and Dutch-speaking part of Belgium participated in the study. No difference between DHH and hearing children were found on general experiences of victimization. However, differences between the groups were found on specific forms of experienced victimization and on the associations between victimization and parental variables. For DHH children, parental sensitivity and parents who challenge their DHH children to become competent in the practical, emotional, cognitive and social domain is associated with them being less victimized. For hearing children at this age these relations were reversed, absent or more complex. Finally, DHH children in special schools were more victimized than DHH children in regular schools. It can be concluded that parents can play an important role in reducing social problems experienced by DHH children and young adolescents.

## Introduction

Deaf or Hard of Hearing (DHH) children might be at greater risk for victimization than hearing youth [1]. Nonetheless, few researchers have been concerned with victimization in this particular group. Chronic peer victimization increases the risk for various problems (e.g., anxiety, depression, and poor academic performance) during childhood and adolescence [2]–[4], but also in adulthood [5]. It is therefore vital to understand the processes that underlie or protect against victimization during childhood and early adolescence. Past literature on hearing children and adolescents has suggested that both the home environment (e.g., parental behavior) and individual aspects (e.g., emotion regulation) are related to peer victimization [6]. Consequently, the present study has two objectives: 1) to examine whether (subsamples of) DHH and hearing youngsters differ in prevalence of self-reported victimization; and 2) to analyze the impact of environmental and individual aspects on victimization among children who are DHH versus hearing children.

### Victimization

Victimization occurs when a child receives negative attention or behavior repeatedly over time from one or more other children [7], [8]. Unfortunately, at least 50% of all school children occasionally experience bully behavior [9]. A risk factor for being bullied is ‘being different’ from the majority [10], [11]. Adding up to this, language difficulties and low levels of socially skilled behaviors have also been associated with being bullied by peers [12], [13]. DHH children can be viewed as being different from the majority because of their observable hearing aids, use of sign language, and/or their distinct speech production. Moreover, DHH children's language problems and impaired socially skilled behaviors have been frequently reported [14]–[16]. All in all, these characteristics raise the question whether DHH children experience more victimization than hearing children. One previous study indeed revealed that DHH children were nominated to be bullied more often than their hearing classmates [15], whereas self-report and parent-report studies failed to confirm this finding [17]–[19]. Besides the fact that these results are mixed, the studies do not provide a full picture of various forms of victimization that can be experienced, in various settings (i.e., beyond the classroom), and among different subsamples of DHH children (e.g., those educated in special versus regular schools or those using signed versus spoken communication).

Another question is whether aspects that are related to victimization are similar in DHH children as compared to hearing children. In theory these should be the same. Alternatively they could be different, because DHH children are growing up in a sound-dominated world with less opportunity to acquire social-emotional knowledge than hearing children. Social-emotional knowledge is, for example, acquired through communicative exchanges between children and their parents [20]. These exchanges between DHH children and their overall hearing parents [21] might be limited due to communication barriers and/or lack of conversational depth and detail [22]. Moreover, DHH children cannot, or to a lesser extent, make use of incidentally learning by overhearing conversations of others. Thus, their auditory deprivation affects the scope of daily learning opportunities about social-emotional functioning and, in turn, may alter associations between victimization and underlying factors typically found in hearing children.

### Aspects associated with victimization

An aspect related to victimization in hearing children is the role of parents [23]–[25]. Parents who are emotionally less stable, overprotective (i.e., treat children younger their age), and less sensitive to their children's needs increase the risk of their children being bullied. No known studies have been conducted on the emotional abilities of parents of DHH children. Additionally, no studies have examined whether parents infantilize their DHH children or have age-appropriate competency expectations. Yet, from research on parents of children with physical disabilities, like chronic illnesses, it is known that they treat children younger their age than parents of typically developing children [26]. Regarding sensitivity, research on very young DHH children (from infancy to early childhood) found parents of DHH children and parents of hearing children to be equally sensitive to their children's needs [27], [28], whereas other findings have implied parents of DHH children to be less sensitive [29]. The interpretation of these outcomes is not straightforward though. A lower score does not necessarily imply that parents are less sensitive. Hearing parents of DHH children are generally limited in their ability to use language as a medium for sharing emotions and experiences with their DHH children [22]. This communication difficulty might prevent these parents from reacting the way they would with hearing children.

Children's ability to regulate their emotions is another important aspect in relation to victimization. Dysregulation of emotions can be observed in heightened levels of negative emotions, such as anger or sadness. Both cross-sectional and longitudinal research has shown that dysregulation of emotions is associated with victimization [30]–[32]. There are indications for emotion dysregulation in DHH children. Compared to hearing peers, DHH children express their anger more openly, and are less likely to communicate their anger strategically to the perpetrator, which could more easily cause escalation of the conflict [33], [34]. Additionally, externalizing problems, such as aggression, and internalizing, withdrawn behaviors are also more often reported in this group than in hearing children [35]–[38].

### Purpose of study

Past studies helped us to acquire basic knowledge about victimization experienced by DHH children. Additional value of our study is the inclusion of a large, heterogeneous sample of DHH children, which enabled us to compare subsamples of DHH children with each other. Furthermore, by using self-report, we were able to examine children's own general experience of victimization in various settings beyond bullying that occurs within the classroom and is seen and rated by classmates. DHH children in special schools are often educated in classes with few children, which make results from peer ratings questionable for this group and difficult to compare with children in regular education. Finally, we explored various forms of victimization rather than simply assessing if children are being bullied or not. DHH children are reported to be neglected more often than their hearing counterparts [39], which could cause differences particularly on items assessing ignorance and exclusion.

This study has two objectives. The first is to compare DHH and hearing participants on prevalence of victimization, and also on levels of parental variables and child variables. In addition, within the DHH sample, we will compare victimization among subsamples based on education type (special versus mainstream education), degree of hearing loss (mild, moderate, or profound), language mode (signed versus spoken language), and hearing device (regular hearing devices versus cochlear implants). A cochlear implant (CI) is a hearing device surgically implanted into the skull, where it converts sounds into electric signals which in turn stimulate the auditory nerve. This nerve leads into the brain, where the sound eventually is ‘heard’.

Second, we will analyze the impact of parental variables and child variables on victimization, and whether the strengths of these relationships differ for children who are DHH or hearing. Indices for parental variables are: parents' emotional intelligence (parents' EI), parental sensitivity, and parents' expectations about the age-appropriate competencies their children should achieve (parents' expectations). Indices for the child variables on emotion dysregulation are their daily levels of anger and sadness. Additionally, communication between parents and DHH children will be assessed to examine whether potential differences between DHH and hearing children on parental variables were attributable to communication characteristics.

In line with findings from studies including parents of children with physical disabilities, we expect parents of DHH children to treat their children younger than their age and therefore have fewer expectations concerning their children's competencies compared to parents of hearing children [26]. Because it is more difficult for hearing parents to share experiences and emotions by means of language with DHH than with hearing children [22], we also expect parents of DHH children to score lower on sensitivity. Additionally, we expect more emotion dysregulation in DHH children as compared to their hearing counterparts [35], [37]. Based on the theory that fewer expectations concerning children's competencies, less sensitive parenting, but more emotion dysregulation are associated with risk of being victimized, we expect DHH children to experience more victimization than hearing children. Concerning differences in victimization between different subsamples of DHH children, the current study is explorative in nature.

A priori there are no grounds to expect any differences between DHH and hearing youth on relations between predictor variables and victimization. Alternatively, as a consequence of DHH children's higher vulnerability in a sound-dominated world, they might require their parents to have fewer expectations concerning certain competencies than hearing children. Additionally, parents' EI might less strongly affect DHH than hearing children, because DHH children lack the acoustic information to interpret the emotional displays and reactions of their parents appropriately. With the present empirical study we aim to unravel these relationships.

We chose the age range of nine to fifteen years old because over this period youngsters face many challenges attributable to biological, developmental, and social changes, which make this a risk period for the development of problems [40]. Some researchers even claim that during this period victimization reaches peak prevalence [32]. Moreover, during this period children make a transition from primary to secondary education. Past research found an effect of school transition on DHH children's well-being [41], which will also be briefly addressed in the current study regarding victimization.

Finally, it should be noted that past studies report gender differences to be less pronounced among victims than among bullies [42]. Though, for completeness, gender will be taken into account both in the scores on victimization as well as in the associations between prediction variables and victimization.

## Methods

### Ethics Statement

The study was approved by the Medical Ethics committee of Leiden University Medical Center, Leiden, the Netherlands, and carried out in accordance with the standards set by the Declaration of Helsinki. Written parental consent was obtained for all children prior to data collection.

### Participants

In total 188 children and adolescents from the Netherlands and the Dutch-speaking part of Belgium were included in the study, of which 94 were DHH and 94 were hearing. An inclusion criterion for the DHH children was to have at least 40 dB hearing loss in both ears (calculated by averaging unaided hearing thresholds at 500, 1000, and 2000 Hertz). Other inclusion criteria were: detection of hearing loss prelingually or perilingually, and no medical or developmental disabilities, such as learning disabilities or autism spectrum disorder. All DHH children were born into hearing families.

The control group of normal hearing children was matched for age, gender, socioeconomic status (SES; based on parental education, occupation and net income), nonverbal IQ, and language comprehension with the DHH group. Inclusion criteria were identical to the DHH group (i.e., no diagnosed disabilities). See Table 1 and Table 2 for specific details of DHH and hearing children. This information was obtained from medical records and parent questionnaires.

**Table 1:** Characteristics of Participants.

	Total sample (N = 188)	
	DHH	Hearing
Number of children - *n* (%)	94	94
Mean age in years (*SD*)	12;02 (1;10)	11;09 (1;04)
Age range in years	9;03–16;00	9;02–14;07
**Sex - ** ***n*** ** (%)**		
Male	48 (51%)	44 (47%)
Female	46 (49%)	50 (53%)
**Questionnaire filled in by - ** ***n (%):***		
Mother/Father/	70 (75%)/17 (18%)/	71 (76%)/15 (16%)/
Both/missing	6 (6%)/1 (1%)	6 (6%)/2 (2%)
**Family composition - ** ***n (%)***		
One-parent/Two-parent/	11 (12%)/83 (88%)/	16 (17%)/76 (81%)/
missing	-	2 (2%)
Socioeconomic status mean (SD)^a^	11.6 (2.2)	11.9 (2.4)
**Ethnicity - ** ***n (%)***		
Both parents Dutch	85 (90%)	78 (83%)
One or both parents other ethnicity	9 (10%)	13 (14%)
missing	-	3 (3%)

a Socioeconomic status score was measured by parental education, occupation, and net income.

**Table 2:** Characteristics specific for the DHH Sample.

	DHH sample (n = 94)
**Degree of hearing loss** a **- ** ***n*** ** (%)**	
Moderate (40–60 dB)	25 (27%)
Severe (61–90 dB)	20 (21%)
Profound (>90 dB)	45 (48%)
missing	4 (4%)
**Preferred mode of communication - ** ***n*** ** (%)**	
Oral language only	72 (77%)
Sign or sign-supported language	22 (23%)
**Type of education - ** ***n*** ** (%)**	
Regular education	65 (69%)
Special education	29 (31%)
**Type of amplification - ** ***n*** ** (%)**	
Hearing aid	56 (60%)
Cochlear implant (CI)	38 (40%)
Mean age of implantation *(range)*	4;01 (1;00–10;08)
Mean number years of CI use (*range*)	8;02 (2;02–13;00)

a Hearing losses of the DHH children were calculated by averaging unaided hearing thresholds at 500, 1000, and 2000 Hertz.

### Materials

The questionnaires used in the present study addressed victimization, parental sensitivity, parents' expectations, parents' EI, and children's levels of sadness and anger. Additionally, communication between parents and DHH children was assessed. Parental sensitivity was drawn from both parent- and child-reports. Parents' expectations and parents' EI were drawn from parent-reports, while children's anger, sadness, and victimization were drawn from children's self-reports. All questionnaires had internal consistencies ranging from sufficient to good, as shown in Table 3. Within the DHH sample, results indicate sufficient to good internal consistency values for the spoken and sign language versions separately (ranging from *α* = .68 to *α* = .88).

**Table 3:** Psychometric Properties of Questionnaires.

	No. of items	Range	Alpha		Means (SD)	
			DHH	H	DHH	H
Victimization	10	1–3	.81	.71	1.48 (.37)	1.45 (.29)
*Parenting variables*						
Parental Sensitivity (parent-report)	10	1–5	.83	.80	4.21 (.45)	4.19 (.41)
Parental Sensitivity (child-report)***	6	1–3	.74	.72	2.61 (.33)	2.78 (.26)
Parents' EI	30	1–7	.85	.74	5.60 (.63)	5.69 (.60)
Parents' Expectations	21	1–3	.83	.77	2.77 (.21)	2.70 (.20)
*Children's emotion dysregulation*						
Anger	4	1–3	.79	.80	1.36 (.39)	1.44 (.45)
Sadness	4	1–3	.86	.81	1.36 (.47)	1.43 (.44)
Communication; parents - DHH children	6	1–3	.70	n.a.	2.48 (.38)	n.a.

*Note.* DHH, Deaf or Hard of Hearing; H, Hearing; EI, Emotional Intelligence; n.a., not applicable.

*** *p<.001*.

### Parent measures

*Parental Sensitivity*, the parent-report version, measures two different parenting behaviors, which according to a prior study by Van Aken and colleagues [43] were found to be associated with children's functioning. The first parenting behavior scale is Responsiveness, which includes four items and reflects the degree to which parents adequately and responsively react to the needs, signals, and conditions of their children (Dutch Parenting Questionnaire [44]). An example item is “I know very well what my child needs and feels”. The second parenting behavior scale is Reinforcement of Good Behavior, consisting of six items, and this scale reflects how often parents praise their children's good behavior. Reinforcement of Good Behavior was derived from the Alabama Parenting Questionnaire [45], [46]. An example item is “I praise my child when he behaves well”. All items could be answered on a 5-point scale ranging from 1 = *never*, to 5 = *always*. The two scales were positively related to each other and correlations with other variables included in this study were in a similar direction. A mean score was calculated over the two scales, indicating Parental Sensitivity.

*Parents' emotional intelligence* was measured with the Trait Emotional Intelligence Questionnaire-Short Form (TEIQue-SF [47]). The TEIQue measures emotional intelligence or emotional self-efficacy, by means of emotion-related behavioral dispositions and self-perceived emotion abilities. The Dutch version [48] consisting of 30 items was used for this study. The participants were asked to respond to the items on a 7-point scale (from 1 = *disagree*, to 7 = *agree*). An example item is: “I often experience difficulties with regulating my emotions”. Some items are negatively formulated and thus reverse scored.

The questionnaire to assess *Parents' expectations* of their children's competencies was based on the Competence model [49] and further developed by a team of developmental psychologists and a child psychiatrist for the purpose of this study. The Competence model describes practical, emotional, cognitive, and social competencies children and adolescents should typically achieve at certain developmental phases. The questionnaire consists of 21 statements. Parents were asked to indicate on a 3-point scale the importance that their child is able to act, or has knowledge about something described by the statement (from 1 = *not important*, to 3 = *important*). An example of a statement is: “I think it is important that my child is able to make appointments on his/her own to play or do activities with friends”.

### Child measures

The questionnaire to assess victimization among children was based on the *Bully/Victim Inventory* [50]. The questionnaire consists of ten items covering physical, verbal, and indirect bullying (see Table 4 for the items). Ignorance and neglect can be a bully experience specific for atypically developing children, such as DHH children [39]. Therefore, the item “Are you invited to birthday parties?” (which was reverse scored) was included to tap into this. The reliability of the questionnaire has been proven in past research [51]. All items could be answered on a 3-point scale (from 1 = *(almost) never*, to 3 = *often*). Prior to completing the questionnaire, the participants were given a definition of bullying, followed by several examples (Appendix S1).

**Table 4:** Items in the Victimization Questionnaire.

1	Are you hit, pushed or kicked?
2	Are you called names?
3	Are mean things said to you? (also by msn, text message, email or social media)
4	Do other children talk viciously about you?
5	Are you laughed at?
6	Are your things/belongings snatched?
7	Are others ignoring you?
8	Are you told that you cannot participate?
9	Do others make you do things, which you actually do not want to do?
10	Are you invited to parties? (*Reverse coded*)

*Parental Sensitivity* from the children's perspectives was assessed with the Self-Esteem questionnaire [52]. This questionnaire measures children's self confidence and social acceptance in several domains, of which the parent domain was used in this study. Participants were instructed to score each item on a 3-point scale (from 1 = *not true*, to 3 = *often true*). An example item is: “My mom or dad makes time to listen to me”.

*Communication* between parents and DHH children was measured with a six item questionnaire developed for the purpose of this study. Children could answer questions on a 3-point scale (from 1 = *(almost) never*, to 3 = *often*). An example item is “My parents look at me, when they want to communicate with me”.

The *Mood questionnaire* [53] comprises four mood scales (three negative: anger, sadness and fear; one positive: happiness). In the present study, only the scales for anger and sadness were used, because these were found to be related to victimization in past literature. Each scale consists of four items. Children were asked “How have you been feeling the past four weeks?” as an introduction to the items, and instructed to score each item on a 3-point scale (from 1 = (*almost*) *never*, to 3 = *often*). Example items are: “I feel angry' and “I feel sad”.

### Indices for children's nonverbal intelligence and language performance

To obtain an indication for nonverbal intelligence of the children, we used two subtests of the *Wechsler Intelligence Scale for Children – Third Edition (WISC-III)*: Block Design (copying small geometric designs with cubes), and Picture Arrangement (arranging pictures to make logical stories) [54], [55]. In a random sample of 23 DHH children, we found high correlations between the present two intelligence subtest scores and earlier assessed complete nonverbal intelligence scores (i.e., *r* = .79, *p*<.001). These tests were either the WISC or the Snijders-Oomen Nonverbal Intelligence Test [56]. We did not obtain IQ normscores for six DHH and four hearing children, due to time constraints.

Children's language performance was assessed with a story- and a sentence- comprehension task. These tasks were used to ensure that children would have sufficient language knowledge to comprehend the items in the psychosocial questionnaires. Hearing children and DHH children using spoken language received the two subtests from the Dutch version [57] of the Clinical Evaluation of Language Fundamentals® - Fourth Edition (CELF® - 4 [58]). DDH children who use sign or sign-supported language received the subtests from the Assessment instrument for Sign Language of the Netherlands [59]. Both tests were comparable with regard to content. Children's language scores were transformed to age-equivalent scores. To 5 DHH and 14 controls the story-comprehension task was not administered, and to 8 DHH and 14 controls the sentence-comprehension was not administered, due to time constraints.

### Procedure

DHH children were recruited from ambulatory care organisations, hospitals, via specific magazines and websites for DHH individuals, and from primary and secondary schools for the deaf and hard of hearing. The group of hearing children was drawn from primary and secondary mainstream schools. These schools were randomly selected, although it was ensured that they accurately reflect the educational system of the Netherlands. The parents of the children received information packages about the study and were invited to participate.

All participants were individually tested at school or at home in two sessions that lasted from 30 minutes to one hour. The two sessions were approximately one week apart. Before actual data collection began, participants were informed that they could request clarification on any item or question from the researcher present. This researcher communicated with the DHH participants in their preferred mode of communication (spoken, sign-supported or sign language). Participants were made familiar with the testing procedure by an introduction and sample questions. Parents could privately fill in their questionnaires through a secured internet survey, or via a paper version that could be sent back to the research group. Information of the children and parents were processed anonymously, but could still be matched by using a unique code for each child.

During data collection, children viewed items one at a time in written Dutch on a laptop, with beneath three response buttons. DHH participants proficient in sign or sign-supported language viewed a video clip of the signed item, in addition to the written Dutch version. Translation from Dutch into sign language was done by a qualified sign language interpreter, after which the items were videotaped, signed by either a deaf individual or a sign language interpreter. Backtranslation did not show divergence between translated and original items.

## Results

### Differences between DHH and Hearing children on Victimization and predictor variables

First, the complete sample of DHH youth was compared to hearing youth on mean levels of Victimization. This revealed no significant difference between the two groups (*t*(186) = −.68; *p* = .50). Further explorative examination of Victimization on item-level by means of a 2 (Group: DHH and hearing)×10 (Victimization items) Multivariate Analysis of Variance (MANOVA) did reveal a multivariate effect for Group (*F*(10,177) = 3.13, *p*<.001, partial *η^2^* = .15). DHH children reported feeling more ignored (*F*(1,186) = 4.77, *p*<.05; partial *η^2^* = .025), received more mean comments (*F*(1,186) = 5.96, *p*<.05; partial *η^2^* = .031), and reported fewer invitations to parties (*F*(1,186) = 5.09, *p*<.05; partial *η^2^* = .027) than their hearing peers.

To explore potential differences on the predictor variables between the complete DHH sample and hearing sample, a 2 (Group: DHH and hearing)×6 (Variables: Parental Sensitivity parent-report, Parental Sensitivity child-report, Parents' Expectations, Parents' EI, Anger, and Sadness) MANOVA was carried out. Results showed a significant multivariate effect for Group (*F*(6,181) = 4.38, *p*<.001, partial *η^2^* = .127), indicating a significant difference between DHH and hearing children. Univariate tests with Bonferroni correction revealed that DHH children, but not their parents, reported a lower score on Parental Sensitivity than hearing children (*F*(1,186) = 14.66, *p*<.001, partial *η^2^* = .07; see Table 3 for the mean values). No other group differences on parental behaviors or on children's levels of Anger and Sadness appeared.

### Difference between subsamples of DHH children

To differentiate within the DHH sample, we ran an Analysis of Variance (ANOVA) in which each of four main effects (i.e., Education type, Hearing device, Degree of hearing loss, and Language mode) on Victimization were explored, with a correction for the remaining three main effects. Results showed a main effect for Education type (*F*(1,85) = 9.29, *p*<.01; partial *η^2^* = .099), while the main effects for Hearing device, Degree of hearing loss, and Language mode were non-significant (*F*(1,85) = 1.31, *p* = .26; *F*(1,85) = .16, *p* = .69; and *F*(1,85) = .62, *p* = .43 respectively). DHH children in Special education (*M* = 1.66, *SD* = .34) reported more Victimization than DHH children in Regular education (*M* = 1.40, *SD* = .36).

Subsequently, for a more comprehensive picture about DHH children's functioning in either mainstream or special education, possible language comprehension differences between the two samples of DHH children were explored. A MANOVA revealed an overall effect for Group (*F*(2,79) = 6.12, *p*<.01; partial *η^2^* = .134). DHH children in Special education had lower Story comprehension (*F*(1,80) = 10.23, *p*<.01; partial *η^2^* = .118) and lower Sentence comprehension (*F*(1,80) = 10.31, *p*<.01; *η^2^* = .102) than DHH children in Mainstream education.

Finally, the effect of Education level (i.e., primary versus secondary schools) on Victimization in DHH children was explored. Results of a *t*-test showed that this effect was not significant (*t*(92) = 1.55, *p* = .12). Two subsequent *t*-tests, in which the DHH sample was divided based on Education type (i.e., mainstream or special), did also not reveal differences on Victimization in DHH children in primary or secondary schools (*t*(63) = 1.67, *p* = .10, and *t*(27) = −.07, *p* = .95, for mainstream and special education respectively).

### Gender differences

*T*-tests were carried out to examine gender differences. These analyses showed no differences on Victimization between boys and girls, both within the total sample (*t*(186) = −.01; *p* = .99), as within the samples of DHH children (*t*(92) = −.38; *p* = .71) and hearing children (*t*(92) = .43; *p* = .67). Furthermore, no differences were found between DHH girls and hearing girls (*t*(94) = −.88; *p* = .38), nor between DHH boys and hearing boys (*t*(90) = −.08; *p* = .94).

### Correlations

#### Parental Sensitivity child-report and Communication in the DHH sample

We examined whether the lower parental sensitivity reported by DHH children compared to hearing children could be due to Communication characteristics between DHH children and their parents. Correlational analyses between the two concepts (Parental Sensitivity and Communication) revealed a significant positive correlation, *r* = .55, *p*<.001.

#### Victimization

Spearman correlations are shown in Table 5. Victimization is negatively correlated with Parental Sensitivity child-report (Parental Sensitivity CR) and positively with Sadness and Anger in both groups. A difference between the groups was also found: only in the DHH group Victimization was negatively correlated with Parents' Expectations.

**Table 5:** Spearman Correlations between Victimization, Parental- and Child variables.

		Parental variables				Child variables	
		2	3	4	5	6	7
1	Victimization	.18/−.17	−.22**	−.07	−.05/−.35**	.22**	.41***
2	Parental Sensitivity (parent-report)	-	.02	.40***	−.10/.21*	−.12	−.05
3	Parental Sensitivity (child-report)		-	.09	.08	−.20**	−.16*
4	Parents' EI			-	.10	−.04	−.05
5	Parents' Expectations				-	−.11	−.14
6	Anger					-	.16/.55***
7	Sadness						-

*Note.* Correlations are provided separately for Hearing and DHH participants when these were found to be significant different (using Fisher Transformation) (Hearing/DHH).

* *p*<.05,

** *p*<.01,

*** *p*<.001.

#### Parent and Child predicting variables

Parental Sensitivity parent-report (Parental Sensitivity PR) was positively related to Parents' EI, while Parental Sensitivity CR was negatively related to children's Anger and Sadness in both groups. Solely in the DHH group significant positive associations were found between Parental Sensitivity PR and Parents' Expectations and between Sadness and Anger.

### Parent and Child variables associated with Victimization

A hierarchical regression analysis with method enter was carried out to evaluate the unique value of parental variables (step 1), child variables (step 2), and interactions between hearing status and parent and child variables (step 3) in predicting victimization. With this final step we were able to assess whether the associations of parental variables and child variables with victimization differ for children who are DHH or who are hearing. Parental variables were entered to the model before child variables. If parental variables lose significant contribution when child variables are added to the model, this might indicate that the parental variables affect the child variables, which in turn affects children's functioning. Also Gender and Age were included in the analyses, but did not make significant contributions; therefore these were omitted from further discussion of results.

**Figure 1:**
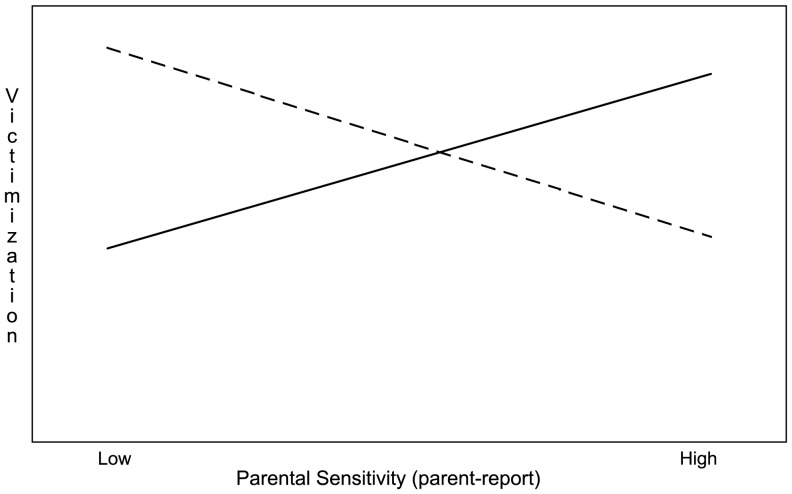
Associations between Parental Sensitivity PR and Victimization for DHH (dotted line) and Hearing Children separately.

**Figure 2:**
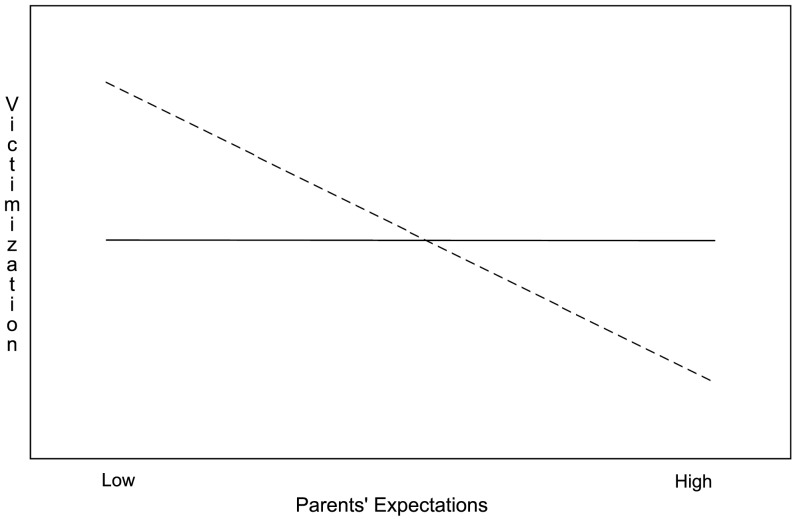
Associations between Parent's Expectations and Victimization for DHH (dotted line) and Hearing Children separately.

As shown in Table 6, in the first step Parental Sensitivity CR and Parents' Expectations were negatively related to Victimization. When the child variables were entered into the model, Parental Sensitivity CR lost significant contribution, whereas Sadness made a significant positive contribution to Victimization. In the final step the interaction terms were entered and this revealed two significant interaction effects: between 1) Hearing Status and Parents' Expectations, and 2) Hearing Status and Parental Sensitivity PR. To examine these interaction effects, we followed the Aiken and West procedure [60] to calculate and plot the effects of Parental Sensitivity PR, and Parents' Expectations on Victimization for both DHH and hearing participants separately. As can be seen in Figure 1, the relation between Parental Sensitivity PR and Victimization is opposite for DHH and hearing children; in the DHH group the relation is negative, and in the hearing group the relation is positive. Furthermore, Figure 2 shows that the relation between Parents' Expectations and Victimization only applies to the DHH group.

**Table 6:** Hierarchical Regression Analysis predicting Victimization from Parental and Child variables.

	Victimization		
	*R^2^*	*Δ R^2^*	*B*
*Step 1*	9.9%		
Hearing status			.05
Parental Sensitivity Parent-report			−.06
Parental Sensitivity Child-report			−.18*
Parents' EI			.05
Parents' Expectations			−.22**
*Step 2*	23.6%	13.7%	
Hearing status			.09
Parental Sensitivity Parent-report			−.01
Parental Sensitivity Child-report			−.11
Parents' EI			.03
Parents' Expectations			−.16*
Anger			.06
Sadness			.37***
*Step 3*	29.4%	5.8%	
Parental Sensitivity Parent-report			.23*
Sadness			.31**
Hearing Status* Parental Sensitivity PR			−.32**
Hearing Status* Parents' Expectations			−.21*

*Note.* Hearing status means DHH or hearing. In Step 3 only significant main- and interaction effects are shown in the table.

* *p*<.05,

** *p*<.01,

*** *p*<.001.

## Discussion

### Victimization

The outcomes of this study showed that DHH children reported victimization as often as their hearing peers, congruent with other studies in which children's own experiences were assessed [17], [18]. The level of hearing loss (moderate, severe or profound) does not effect experience of victimization, nor does the type of hearing device (CI or traditional hearing devices) or language mode (sign supported versus spoken language).

Nevertheless, the DHH children in special education reported victimization more often than DHH peers in regular education. Possibly, outside school in their own neighborhood, these children are a target of victimization because they are different by attending a special school, or because of communication difficulties between them and the hearing children next door. It could also be that in the special classes, DHH children of various levels of intellectual, linguistic, and social emotional abilities are placed together, creating large differences between children and therefore increasing the risk for victimization [61]. Additionally, children in special schools experience more difficulties than children in regular education, most likely due to these students' special characteristics and not the education type itself [62]. This discrepancy is exemplified by our observation of significant lower levels of language competence in DHH children attending special education compared to DHH children attending mainstream schools. Relatively lower levels of language competence have been found to be related to poor peer relationship quality [63].

Additionally, differences between the overall samples of DHH and hearing children occurred when they were compared on item-level victimization. DHH children reported fewer invitations to parties, received more mean comments, and being more often ignored than hearing children. Thus, although the overall results are positive, parents, teachers and/or professionals working with DHH children should bear in mind that problems in specific areas may be present in order to enhance positive peer interactions between DHH children and their (DHH or hearing) peers.

### Parenting

Parents of DHH and hearing children reported equal expectations concerning their children's competencies and equal levels of sensitivity towards their child. However, DHH children reported their parents to be less sensitive than hearing children. DHH children reported, more often than their hearing peers, that their parents make less time to listen to them and value them less important. Previous studies stated that parents ask their DHH children less often about their school day or plans for the coming day, and talk less with them about their friends than parents of hearing children [64]. Thus, the reported lower parental sensitivity may be the result of communication-related facets of parenting behaviors, rather than parents being less caring or sensitive. This is underlined by the fact that we found an association between parental sensitivity and communication between parents and DHH children, such that a higher score on communication was related to more parental sensitivity. Another argument supporting a communication-related explanation is the fact that DHH and hearing children equally often report that their parents do nice activities with them.

### Emotion dysregulation: levels of sadness and anger

Contrary to our expectations, no differences were reported between DHH and hearing children on overall mean levels of sadness and anger, implying compatible levels of emotion regulation in both groups. Yet, results from the bivariate correlational analyses showed that the correlation between anger and sadness is higher in DHH children than within the hearing sample (i.e., *r* = .55 and *r* = .16, respectively. Refer to Table 5). Possibly, DHH children have more difficulty in discriminating between emotions within the negative spectrum. Previous findings [65] suggest similar patterns. The emotional functioning of DHH children therefore remains an area worth considering in future research. Future directions must move beyond simply mean scores, and consider associations between variables.

### Variables associated with victimization

With this study we also examined how parental and child variables were related to victimization in hearing versus DHH children. For both groups, the overall picture emerged that child-reported parental sensitivity is associated with children being bullied, and can be measured by their level of sadness. This is in line with previous research, which has suggested that parenting styles can affect children's ability to regulate their emotions, which in turn affects children's social adjustment [66], [67].

Two important differences appeared between the groups regarding the associations between parental variables and victimization. First, parents' expectations were related to less victimization in DHH but not in hearing children. Parents who have fewer expectations regarding their children's competencies may restrict children's exposure to daily life challenges. This, in turn, interferes with children's opportunities to become independent and assertive individuals, and to learn interpersonal skills [68], [24]. The absence of this relation between parents' expectations and victimization in hearing children could be due to the fact that the items used in this study are age-appropriate for typically developing children. For example, traveling by public transport, making appointments with friends, or do some shopping on their own, are behaviors that hearing twelve-year-olds perform on a daily basis with minimal parental involvement. Yet, DHH children who are less independent and thus require more encouragement from their parents are also the children that are bullied more often.

Second, sensitivity by parents towards their children's needs was related to less victimization in the DHH group, but the opposite was true for the hearing group. It is thought that parents who are sensitive, and regularly adjust their responses to their children's needs and behaviors, communicate a sense of interest and involvement. They also provide children with feedback that may allow them a sense of control and influence over others [24] and thus, over peers and possible bullies. This hypothesis is supported by the child-reports about parents' sensitivity, and by parent-reports of parents of DHH children. Only parent-reports of sensitivity towards their hearing children revealed an opposite pattern: more parental sensitivity was related to more victimization in hearing children. Possibly, the sensitivity as it was measured by parents towards their hearing children is more appropriate and adaptive for a younger age group. Showing these behaviors at the current older age range might indicate that parents interfere too much with their hearing children's independence, which in turn makes them more vulnerable to victimization. For DHH children, these sorts of directive parents' behaviors appear to be adaptive at the age of 9 to 15. Future studies could perhaps include different age groups to compare what kinds of parental support and involvement is required for DHH and hearing children at different stages in their lives.

This recommendation also counts for the association between parents' emotional intelligence and children's victimization. For both groups, this association was not found in the current study. Possibly, the mechanism of modeling by which parents can influence their children [69], [70] is applicable when children are at a younger age.

### Implications for future research

Several implications for future research are given throughout the discussion. We would like to add that future studies concerning bully behavior among peers could include peer nominations and/or observational measures. Although we believe that self-reports are appropriate, including other-reports would enable comparison between subjective and objective experiences of victimization. In the current study we were also not aware of the parental experience of communication with their DHH child and how this compares to the child's experience. Future research could include both parent- and child-reports to assess communication from different viewpoints. A related topic worth in-depth examination is how communication between parents and children affects the influence parents have on their children. It is known that successful exchange (regardless of modality) of ideas and information between parents and children is critical for the overall development in DHH youth [71], [72]. However, an association between communication and children's functioning is dissimilar from the question how communication affects parenting behaviors and, in turn, children's functioning. Results of the current study showed a relation between parent-DHH child communication and parenting behavior, yet, more careful exploration is required. Future studies should include a multimethod approach in which both parents and children are questioned and observed in their interactions.

Future research could also explore bidirectional and reciprocal associations between parenting styles and individual differences among children. Parents can affect their children's behavior, but children also react in ways that elicit certain parenting behaviors [73]. For example, the positive association between parental sensitivity and victimization in their hearing children could alternatively indicate that parents are trying hard to be sensitive and to listen well to their victimized children. Longitudinal research in particular would enable examination of causality. Causal directions of relationships should be explored not only between parents and children, but also, for example, regarding the currently found association between children's sadness and victimization. This will unravel whether children's sadness makes them more vulnerable to being victimized or whether being victimized increases children's sadness.

Larger samples would make it possible to examine associations between predictor variables and victimization within the heterogeneous group of DHH children. Future research could include more children who use sign or sign supported language in particular (*n* = 1 and *n* = 21, respectively in this study). DHH children from deaf parents could additionally be included. Although this group is only about 5% of the DHH population [21], including DHH parent-child dyads would shed more light on the relationship between DHH children and their parents. Finally, in our sample no difference was found between DHH children with regular hearing devices and children with CI. In general, the CI children in our study are implanted at an older age (mean age = 4;01 years) than children are nowadays. Future research could focus on this rapidly growing group of early implanted children.

### Conclusion

Overall, DHH children do not report to be victimized more often than hearing children, although a distinction should be made between DHH children in mainstream and special education. DHH children do report some forms of victimization more often than hearing children. Victimization should therefore not be neglected in DHH youth. The current research shows that parents can be included in intervention programs for reducing victimization in DHH children at the age range of 9 to 15 years. Parents who are sensitive towards their DHH children and challenge them to become competent in the practical, emotional, cognitive and social domain decrease their children's chance to be victimized.

## Supporting Information

Appendix S1 **Definition and Examples of Bully Behavior.**
(DOC)
