# Supplementary material for: Peer Victimization Experienced by Children and Adolescents Who Are Deaf or Hard of Hearing
Source: PLoS One. 2012 Dec 19;7(12):e52174. doi: 10.1371/journal.pone.0052174 (PMC3526587; doi:10.1371/journal.pone.0052174)
Supplement: Appendix S1 — Definition and Examples of Bully Behavior. (DOC) [file pone.0052174.s001.doc]

Bullying is: again and again, on purpose, being mean to someone to hurt him or her. Or to make that person sad.

Examples of bully behavior are:

- laughing at or about somebody

- pushing, hitting or kicking someone

- scaring or threatening someone

- taking away things from someone.

Bullying can also be when you ignore a person, such as:

- telling someone to go away

- pretending you do not see that person

Bullying can occur in the streets, but you can also send a nasty text message with your mobile. Or you can block someone with msn.

Are you being bullied sometimes? Remember that your answers remain secret.

The next questions are about other children who are bullying YOU.

[Followed by the items containing hurtful acts towards them]

*Note*: This instruction is based on work by and personal conversation with Frits Goossens.
